# A meta-analysis of the haemodynamics of primary hypertension in children and adults

**DOI:** 10.1097/HJH.0000000000003326

**Published:** 2022-12-02

**Authors:** Ye Li, Emily Haseler, Ryan McNally, Manish D. Sinha, Phil J. Chowienczyk

**Affiliations:** a King's College London British Heart Foundation Centre, St. Thomas’ Hospital, Westminster Bridge; b Evelina Children's Hospital, London, UK

**Keywords:** cardiac output, haemodynamics, hypertension, systemic vascular resistance

## Abstract

We performed a systematic review and meta-analysis to determine the relative contributions of elevated cardiac output and systemic vascular resistance to hypertension in children and adults. This included 27 studies on 11 765 hypertensive and normotensive children and adults in whom cardiac output was measured. Cardiac output but not systemic vascular resistance was elevated in hypertensive compared to normotensive children and young adults (difference in means 1.15 [0.78–1.52] l/min, *P* < 0.001). In older hypertensive adults, both were elevated compared to normotensive individuals (0.40 [0.26–0.55] l/min, *P* < 0.001 and 3.21 [1.91–4.51] mmHg min/l, *P* < 0.001 for cardiac output and systemic vascular resistance, respectively). The main haemodynamic alteration in primary hypertension (including obesity-hypertension) in both children and young to middle-aged adults is an elevation of cardiac output. With longer duration and greater severity of hypertension there may be progression from a ‘cardiac’ to a ‘vascular’ phenotype with increased systemic vascular resistance.

## INTRODUCTION

Mean arterial blood pressure (MAP) is determined by the product of cardiac output (CO) and systemic vascular resistance (SVR). Thus, in hypertension, where MAP is usually elevated because of an increased systolic or diastolic blood pressure or both, this component of hypertension can be attributed to increased CO, increased SVR or both. Early studies measuring CO by the dye dilution method in adults with severe hypertension demonstrated reduced CO and increased SVR compared to normotensive individuals [1,2]. Later studies in adults with established hypertension performed mainly in the 1980s confirmed these findings, with increased SVR (or SVR indexed to body surface area, SVRI) as the characteristic finding [3–5]. This has led to the view that primary hypertension is predominantly a ‘vascular’ rather than ‘cardiac’ problem. However, in hypertensive children and in young adults with borderline and mild hypertension, a hyperkinetic circulatory state with raised CO has been described [6–9]. A hyperkinetic state has also been described in adults with primary hypertension [10]. In older individuals, raised systolic blood pressure and isolated systolic hypertension (ISH) have been attributed to increased aortic stiffness rather than increased SVR [11,12]. Thus, the major haemodynamic alterations that contribute to the majority of cases of primary hypertension in the general population remain unknown.

In the present study, we perform a systematic review and meta-analysis of previous studies comparing measures of CO and SVR in patients with established hypertension with those in normotensive control individuals to determine whether primary hypertension is characterized predominantly by elevated CO or elevated SVR. Where available we also compare measures of aortic stiffness in hypertensive and normotensive individuals.

## METHODS

The systematic review and meta-analysis was carried out in accordance with PRISMA (Preferred Reporting Items for Systematic Reviews and Meta-Analysis) guidelines [13] and was registered on PROSPERO (ID: CRD42020217927). A systematic literature search was performed on four databases; Ovid MEDLINE(R) Epub Ahead of Print, In-Process & Other Non-Indexed Citations, Ovid MEDLINE(R) Daily and Ovid MEDLINE(R), (1949 to 20 September 2020), PubMed, (1946 to 21 September 2020), EMBASE (1974 to 2020 week 38), and The Cochrane Central Register of Controlled Trials (CENTRAL) databased (up to 21 September 2020). We also manually searched the references of all relevant publications. Studies that were included were those examining hemodynamic measures including cardiac output (CO), systemic vascular resistance (SVR), and pulse wave velocity (PWV) in hypertensive and normotensive individuals. The keywords used included ‘hypertension’, ‘blood pressure’, ‘cardiac output’, ‘cardiac index’, ‘stroke volume’, ‘pulsatile flow’, ‘blood flow velocity’, ‘vascular stiffness’, ‘systemic vascular resistance’, and ‘total peripheral resistance’. Medical subject headings (MeSH) and non-MeSH terms were used to search the databases for relevant publications. The search was limited to the English language and full-text only articles. The full search strategy for PubMed is provided in the supplementary material, Supplemental Digital Content.

### Study selection and eligibility criteria

Papers were initially screened by title and abstract. Studies were eligible for inclusion if they contained *in vivo* human data; provided direct haemodynamic measures and were not review papers, meta-analyses, commentaries, or editorials; provided data on both normotensive individuals and individuals with primary hypertension (with hypertension defined as SBP ≥140 mmHg and/or DBP ≥90 mmHg, or previously diagnosed hypertension and/or prescription of antihypertensive therapy); had a minimum of blood pressure (systolic and diastolic blood pressures, or mean arterial blood pressure), cardiac output and systemic vascular resistance measured/calculated. Exclusion criteria were studies on prehypertension or borderline hypertension; studies in patients with known or suspected secondary hypertension; studies in patients with a history of established cardiac or vascular diseases other than hypertension; studies on clinical trials of antihypertensive agents; mean age difference between normotensive and hypertensive groups >10 years. Study selection, data collection and quality assessments were conducted by two reviewers independently. Disagreements were resolved by consensus and, if needed, consultation with a third reviewer.

### Data extraction and processing

Data were extracted independently using a standard form in an Excel spreadsheet. This included: first author, year of publication, sample size, ethnicity, average age, sex distribution, body mass index (BMI), definition of hypertension, use of antihypertensive drugs (if available), washout period, systolic blood pressure (SBP), diastolic blood pressure (DBP), mean arterial pressure (MAP), and pulse pressure (PP), heart rate (HR), stroke volume (SV), CO, SVR, and PWV and the measurement technique for CO. PP was calculated as the difference of SBP−DBP and MAP as DBP + 1/3 PP if these values were not reported directly. CO and SVR were calculated from SV and HR or from indexed values and body surface area (BSA) if not reported directly. All measurements were summarized as mean ± standard deviation (SD), with SD calculated using the method presented by Wan *et al.*[14] from median and interquartile range if these values were reported rather than mean and SD. If values for the two sexes or other subgroups were reported separately, values for the subgroups were combined into a single group, with means calculated as a weighted average and SD calculated using the formulas presented in the Cochrane Handbook [15]. For example,

(1) $$\text{SD}=\sqrt{\frac{n_{\text{m}}\cdot \text{S}{\text{D}}_{\text{m}}^{2}+n_{\text{f}}\cdot \text{S}{\text{D}}_{\text{f}}^{2}}{n_{\text{m}}+n_{\text{f}}}},\;$$

where *n*_m_ and *n*_f_ are the sample sizes of male and female groups, and SD_m_ and SD_f_ the standard deviations of values for the male and female sub-groups. In cases where mean values of CO were calculated as mean SV × mean HR or mean CI × mean BSA the covariance of the components of the product was taken as zero leading to a small underestimation in the calculated CO (since the average of a product = product of averages plus covariance of the component variables of the product [16]). In these cases, the SD of CO was calculated using the error propagation for multiplication and division [17]. For example,

(2) $$\text{SD}=\text{CO}\cdot \sqrt{{\left(\frac{\text{S}{\text{D}}_{\text{SV}}}{\text{SV}}\right)}^{2}+{\left(\frac{\text{S}{\text{D}}_{\text{HR}}}{\text{HR}}\right)}^{2}},\;$$

where SD_SV_ and SD_HR_ are the SDs of SV and HR, respectively. Units of CO and SVR were converted to l/min and mmHg min/l, respectively, if other units were used.

### Quantitative data synthesis and statistical analysis

Comprehensive Meta-Analysis Software Version 3 (Biostat, Englewood, New Jersey, USA) was used to perform the meta-analysis. Net differences in CO, HR, SV, SVR, and PWV were obtained as the difference in values between hypertensive and normotensive individuals. Results were calculated separately for children and young adults (age < 35 years) and for older adults (age ≥ 35 years) as well as an overall result for all age groups. A random-effects model was used to compensate for between-study heterogeneity in characteristics, with calculation of the mean differences in CO, HR, SV, SVR and PWV, and their 95% confidence intervals (CIs). Statistical heterogeneity was assessed with the Cochran's *Q* test. *P* < 0.05 was considered statistically significant and all tests were two-tailed. Random-effects meta-regression was performed using the method of moments to evaluate the association between mean differences in CO and SVR in hypertensive and normotensive groups with BMI, with the severity of the blood pressure assessed as the mean difference of MAP and PP between the hypertensive and normotensive groups and with the mean age of individuals.

### Assessment of risk of bias

Characteristics and quality of the included studies were evaluated by the Newcastle−Ottawa scale. Studies are considered of good quality if the total score is at least 6/9. Potential publication bias was assessed by inspection of Begg's funnel plot asymmetry and Egger's asymmetry tests [18].

## RESULTS

**FIGURE 1:**
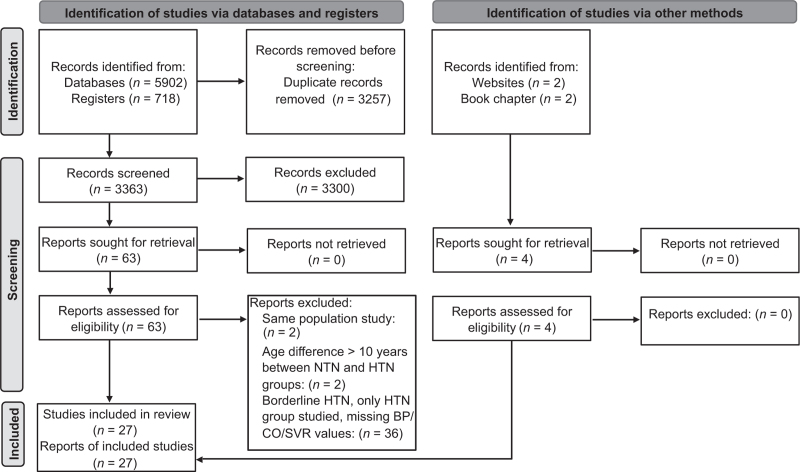
Flowchart of selection of studies for the systematic review based on predefined search strategies and inclusion/exclusion criteria. HTN, hypertensive; NTN, normotensive.

The study selection process is detailed in a flow chart as per PRISMA guidelines (Fig. 1). The initial PubMed search returned 4140 results, MEDLINE 893, EMBASE 869, and Central 718 (6620 in total). After removal of duplicates there were 3363 articles of which 3300 were excluded based on title and abstract. The remaining 63 articles were assessed for eligibility and 40 were excluded for various reasons (Fig. 1). The remaining 23 articles and four articles from manual searches were included in the qualitative synthesis to give a total of 27 studies comprising 11 765 individuals including studies in children and adolescents (*n* = 741), young adults (*n* = 6497) and older adults (*n* = 4527).

### Characteristics of individuals in the selected studies

Data extracted from the included studies is summarized in Table S1, Supplemental Digital Content. The mean age of hypertensive individuals in each study ranged from 11 to 75 years and in most studies was similar to that of comparator normotensive individuals. Only three of 27 of the studies provided separate data for male and female sex [19–21]. Ethnicity was reported in seven of 27 of the studies [8,21–26], with no details of differences in haemodynamic measurements between hypertensive and normotensive groups reported according to ethnicity. The sub-type of hypertension (isolated systolic hypertension, isolated diastolic hypertension, systolic–diastolic hypertension) was provided in only two of 27 of the studies [8,23]. In the majority of studies (16/21) where treatment information was available, hypertensive individuals were either untreated or treatment was washed out for at least 1 week (range 1–8 weeks). The mean difference in BMI between hypertensive and normotensive subjects ranged from 1.8 to 6.6 kg/m^2^ in children and young adults, and from 0.08 to 5.1 kg/m^2^ in older adults. The mean difference in BP between hypertensive and normotensive individuals at the time of study ranged from 8.4 to 27.9 mmHg for MAP in children and young adults, and from 7.4 to 56 mmHg for MAP in older adults. CO was measured by the cardiogreen method in four of 27 [1,2,27,28], cardiac catheterization in two of 27 [29,30], gas rebreathing in one of 27 [21], an oscillometric method in three of 27 [24,31,32], by MRI in four of 27 [8,33–35], by volume-clamp photoplethysmography in one of 27 [23], and by echocardiography in the remaining 12 of 27 studies. Eighteen of 27 studies provided absolute measures of CO (15/18 studies) or absolute measures of SV (3/18 studies [8,23,36]) from which CO was calculated. In the other nine of 27 studies [1,2,19,26,28,29,33,37,38] mean values of CI and SVRI were converted to CO and SVR as detailed in methods. Unadjusted values of CO were reported in all but one study [8] where values were adjusted for differences in age, sex and ethnicity between hypertensive and normotensive groups. Aortic stiffness estimated by carotid-femoral PWV was provided in eight of 27 studies [20,21,24,31,32,35,36,39,40], and by aortic PWV in three studies [23,32,34].

### Meta-analysis: difference in haemodynamic characteristics between hypertensive and normotensive groups

**FIGURE 2:**
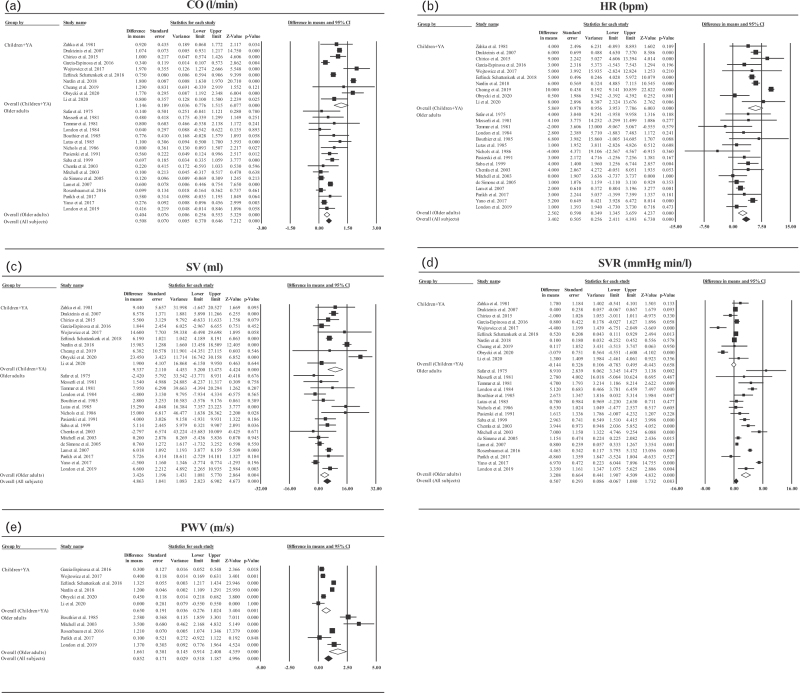
Forest plots displaying difference in means and 95% confidence intervals (CIs) between hypertensive and normotensive groups in cardiac output (CO), heart rate (HR), stroke volume (SV), systemic vascular resistance (SVR), and pulse wave velocity (PWV). Black squares indicate difference in means in individual studies, white diamonds mean value of difference in means in children and young adults (YA) and in older adults, black diamonds indicate overall (individuals in all age groups) mean value of difference in means.

CO was higher in hypertensive compared to normotensive groups both in children and young adults and in older adults (overall difference in means 1.15 [95% CI, 0.78–1.52] l/min, *P* < 0.001) and 0.40 [0.26–0.55] l/min, *P* < 0.001) for children and young adults, and older adults, respectively). This was driven by greater HR and SV in hypertensive children and young adults (difference in means 6 [4–8] bpm and 9.34 [5.20–13.5] ml for HR and SV, respectively, each *P* < 0.001) and greater HR and SV (difference in means 3 [1–4] bpm, *P* < 0.001 and 3.43 [1.08–5.77] ml, *P* = 0.004 for HR and SV, respectively) in older hypertensive adults compared to values in corresponding normotensive groups. In children and young adults there was no significant difference in mean values of SVR between hypertensive and normotensive groups (difference in means −0.14 [−0.78–0.50] mmHg min/l, *P* = 0.66). In older adults, the difference in SVR between hypertensive and normotensive groups was significant (difference in means 3.21 [1.91–4.51] mmHg min/l, *P* < 0.001). PWV was higher in hypertensive compared to normotensive groups in both children and young adults (difference in means 0.65 [0.28–1.02] m/s, *P* = 0.001) and in older adults (difference in means 1.66 [0.91–2.41] m/s, *P* < 0.001, Fig. 2). Heterogeneity of the results (difference in haemodynamic measures between hypertensive and normotensive groups) across studies was at least moderate (*I*^2^ > 25%, each *P* < 0.02). No trend with respect to the date in which studies were performed was seen (see Figure S1, Supplemental Digital Content in which results are listed by date rather than by age of individuals).

### Meta-regression: relation of differences in haemodynamic measures to differences in BMI, severity of hypertension and age of study group

**FIGURE 3:**
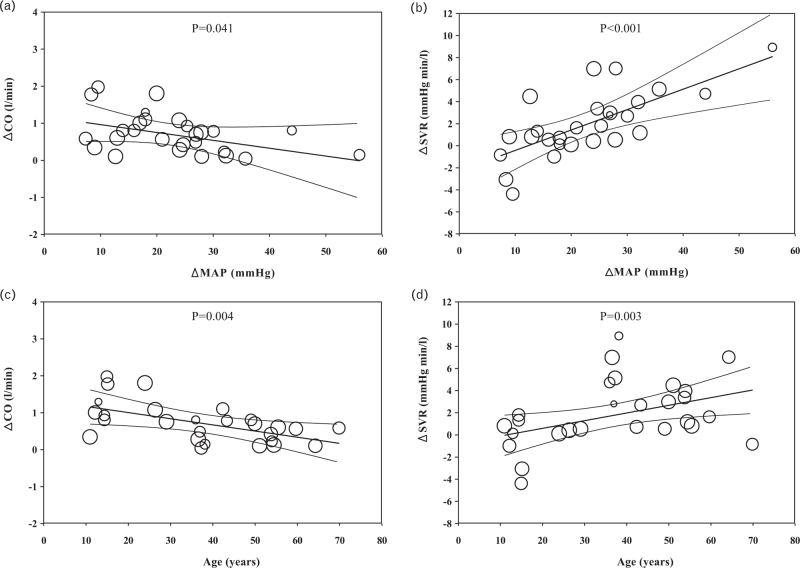
Meta-regression of the association between difference in means for cardiac output (ΔCO) and systemic vascular resistance (ΔSVR) between hypertensive and normotensive groups versus difference in mean arterial pressure (ΔMAP, panels a and b) and versus mean age of the study groups (panels c and d). *P* values indicate the significance of the meta-regression.

Random-effects meta-regression was performed to examine whether heterogeneity in the difference in CO, HR and SV between hypertensive and normotensive groups related to corresponding differences in BMI. There was a trend to a positive relationship between difference in HR and difference in BMI (*β* = 0.24, *P* = 0.16) between hypertensive and normotensive groups and between group differences in SV and BMI (*β* = 0.15, *P* = 0.08). Difference in CO was significantly related to difference in BMI (*β* = 2.21, *P* = 0.03) between hypertensive and normotensive groups. Results of meta-regression analysis to examine whether heterogeneity in the difference in haemodynamic measures related to the severity of hypertension as assessed by the difference in BP between hypertensive and normotensive groups are shown in Table S2, Supplemental Digital Content. Difference in CO between hypertensive and normotensive groups was negatively associated with difference in MAP between the groups, whereas that for SVR was positively associated with the difference in MAP (Fig. 3). When examining how the contribution of CO and SVR to hypertension varied with mean age within each study, there was a negative association of the difference in CO between hypertensive and normotensive groups with age and a positive association of that of SVR with age (Fig. 3).

### Assessment of risk of bias

Table S2, Supplemental Digital Content reports Newcastle−Ottawa scale scores and quality assessment of the included studies; all studies scored six or more, indicating good quality and low risk of bias. The funnel plot of standard error vs. effect size was asymmetric and suggestive of potential publication bias (Figure S3, Supplemental Digital Content). However, presence of publication bias was not indicated by Egger's linear regression (*P* > 0.1). After adjustment of effect size for potential publication bias using the ‘trim and fill’ correction, no additional studies were imputed for CO, HR, SV, SVR and PWV and point estimates and 95% CI of the results for the combined studies were unchanged.

## DISCUSSION

To our knowledge, this is the first systematic review and meta-analysis to assess the major haemodynamic changes associated with hypertension. Our findings confirm that in children, the primary alteration is an increase in CO. They show that this remains the main alteration in young adults with hypertension and that CO is still elevated in older adults with hypertension compared to values in normotensive adults. We observed no significant difference in SVR between hypertensive and normotensive children and young adults. In older adults, the difference in SVR between hypertensive and normotensive groups was significant but there was a high degree of heterogeneity between studies. In meta-regression, we found that the contributions of CO and SVR to hypertension varied progressively with age, with CO being more important in younger individuals and SVR in older individuals. We also found that the difference in SVR between hypertensive and normotensive groups was related to the severity of hypertension as measured by the BP difference between hypertensive and normotensive group and there was a negative association for the difference in CO between hypertensive and normotensive groups and severity of hypertension. The present findings can, therefore, be reconciled with early but carefully conducted studies on the haemodynamics of hypertension in adults in which an increase in SVR was identified as the primary abnormality since these studies were performed mainly in adults with severe hypertension [1,2,20].

The transition of a predominantly ‘cardiac’ hypertensive phenotype to a ‘vascular’ phenotype with age and severity of hypertension are consistent with the concept that hypertension of longer duration and/or more severe hypertension is associated with arterial remodelling to increase SVR. Eutrophic or hypertrophic remodelling of the resistance arterioles in human hypertension and experimental models of hypertension is well recognized and, as with cardiac remodelling, probably relates to the severity and duration of hypertension [41]. Both types of remodelling lead to a reduced lumen diameter and therefore to an increased SVR. At the same time cardiac remodelling associated with hypertension of greater severity and longer duration could result in some impairment of cardiac function offsetting the primary haemodynamic tendency to an increased CO in earlier stages of hypertension.

An important consideration when assessing haemodynamic properties such as CO and SVR is whether these should be normalized for size. Most of the hypertensive groups in our study had higher mean BMI compared to their comparator normotensive groups. However, correction of CO for adiposity (or other aspects of body composition) is not appropriate when addressing the question as to whether the primary haemodynamic abnormality in hypertension is an increase in CO that may be in part secondary to adiposity/body composition. In meta-regression analysis, we found a modest association of the difference in CO between hypertensive and normotensive groups with the corresponding difference in BMI which is consistent with at least some of the cardiac overactivity being driven by adiposity.

The main focus of the present study is the relative contributions of CO and SVR to hypertension. However, it should be emphasized that CO and SVR determine only MAP. PP (which for a given diastolic pressure will determine systolic pressure) is determined by more complex ventricular−vascular interactions including aortic stiffness Aortic stiffness determines the reservoir properties of the arterial tree and may also influence pressure wave reflections [12]. Aortic stiffening could, therefore, account for isolated systolic hypertension in the absence of an increase in CO or SVR. In the present analysis, we found aortic stiffness as measured by PWV to be elevated in hypertensive compared to normotensive groups. There was a positive association between the difference in PWV between hypertensive and normotensive groups and severity of hypertension as assessed by difference in MAP between groups. These results are consistent with the known bidirectional relationship between PWV and hypertension and with PWV being elevated either as the result or cause (or both) of hypertension [42].

The role of increased cardiac activity (increased heart rate and stroke volume) and CO as the major haemodynamic determinant of primary hypertension in young to middle-aged persons has potential implications for the optimal treatment of primary hypertension. If this starts with cardiac overactivity, with a later increase in SVR due to arterial remodelling or other factors, then consideration should be given to interventions that may reduce cardiac overactivity. Specific treatments to reduce sympathetic drive and volume overload (which might reduce CO via effects on heart rate and SV) might prevent hypertension from progressing to the phase characterized by increased SVR. It is notable that obesity hypertension is thought to be characterized by both sympathetic activity and volume overload [43,44]. Weight reduction, therefore, could be particularly important in normalizing hypertension in younger individuals. The relative benefits of different types of treatment could differ according to age and duration of treatment as hypertension transitions from a cardiac to vascular phenotype.

Strengths of the systematic review and meta-analysis include the systematic literature search using multiple databases based on a priori criteria to minimize publication bias, and use of well established meta-analysis procedures for our analysis. Limitations include the limited characterization of the hypertensive and normotensive groups, particularly with regard to ethnicity, hypertension sub-type (e.g. isolated systolic hypertension) and treatment. The majority of studies were performed on individuals who were untreated or in whom treatment was withdrawn for at least 7 days but residual effects of treatment might have been present. In a minority of studies mean values of CO were calculated from the product of the mean of CI and BSA, which would have led to a small underestimation of mean CO. Whether there is a transition within individual hypertensive individuals from a state characterized by cardiac overactivity at a young age to that characterized by increased SVR and less cardiac overactivity at older age would ideally be tested in longitudinal studies. However, it is not possible to perform such studies in un-treated individuals. Finally, our analysis focused on MAP rather than PP. However, MAP is highly predictive of outcomes in individuals of all age and is the component of BP mostly strongly related to outcomes in young to middle-aged individuals [45]. Further work is required to determine the more complex haemodynamic determinants of a difference in PP between hypertensive and normotensive individuals. Finally, whilst the results of this meta-analysis are representative of mean values within normotensive and hypertensive individuals within the population, individual children and adults with hypertension may present a range of phenotypes across the spectrum of increased resistance to increased cardiac output.

In summary, this meta-analysis suggests that the main haemodynamic alteration in primary hypertension in both children and young adults is an elevation of cardiac output. At older age and with (presumably) longer duration and greater severity of hypertension there is progression from this ‘cardiac’ to a ‘vascular’ phenotype with increased systemic vascular resistance and a less pronounced elevation of cardiac output, possibly due to arterial remodelling and due to relative compromise of left ventricular function. In children and young adults, interventions targeted at reducing cardiac overactivity might prevent progression to the ‘vascular’ phenotype associated with more severe hypertension in older adults.

In conclusion, the main haemodynamic alteration in primary hypertension (including obesity-hypertension) in both children and young to middle-aged adults is an elevation of cardiac output. With longer duration and greater severity of hypertension there may be progression from a ‘cardiac’ to a ‘vascular’ phenotype with increased systemic vascular resistance. Interventions targeted at reducing cardiac overactivity in children and young adults might prevent progression to the ‘vascular’ phenotype associated with more severe hypertension in older adults.

## ACKNOWLEDGEMENTS

This research was supported by a British Heart Foundation Project Grant PG/17/50/32903. This work also received support from the National Institute for Health Research Facility and Biomedical Research Centre based at Guy's and St. Thomas’ NHS Foundation Trust and King's College London.

### Conflicts of interest

There are no conflicts of interest.

## Supplementary Material

Supplemental Digital Content
